# Effects of interleukin-1 antagonism and corticosteroids on fibroblast growth factor-21 in patients with metabolic syndrome

**DOI:** 10.1038/s41598-021-87207-w

**Published:** 2021-04-12

**Authors:** Fahim Ebrahimi, Sandrine Andrea Urwyler, Matthias Johannes Betz, Emanuel Remigius Christ, Philipp Schuetz, Beat Mueller, Marc Yves Donath, Mirjam Christ-Crain

**Affiliations:** 1 Division of Endocrinology, Diabetes, and Metabolism, University Hospital Basel, Basel, Switzerland; 2 Department of Clinical Research, University Hospital Basel, Basel, Switzerland; 3 Division of Endocrinology, Diabetes, and Metabolism, University Department of Medicine, Kantonsspital Aarau, Aarau, Switzerland; 4 University Center for Gastrointestinal and Liver Diseases, University Hospital Basel, Basel, Switzerland

**Keywords:** Chronic inflammation, Obesity, Metabolic syndrome

## Abstract

Fibroblast growth factor-21 (FGF21) is elevated in patients with the metabolic syndrome. Although the exact underlying mechanisms remain ill-defined, chronic low-grade inflammation with increased Interleukin-(IL)-1β expression may be responsible. The aim of this study was to investigate effects of two different anti-inflammatory treatments (IL-1 antagonism or high-dose corticosteroids) on FGF21 in patients with the metabolic syndrome. This is a secondary analysis of two interventional studies in patients with obesity and features of the metabolic syndrome. Trial A was an interventional trial (n = 73) investigating short-term effects of the IL-1 antagonist anakinra and of dexamethasone. Trial B was a randomized, placebo-controlled, double-blinded trial (n = 67) investigating longer-term effects of IL-1 antagonism. In total, 140 patients were included in both trials. Median age was 55 years (IQR 44–66), 26% were female and median BMI was 37 kg/m^2^ (IQR 34–39). Almost half of the patients were diabetic (45%) and had increased c-reactive protein levels of 3.4 mg/L. FGF21 levels correlated with fasting glucose levels, HOMA-index, C-peptide levels, HbA1c and BMI. Short-term treatment with anakinra led to a reduction of FGF21 levels by − 200 pg/mL (95%CI − 334 to − 66; p = 0.004). No effect was detectable after longer-term treatment (between-group difference: − 8.8 pg/mL (95%CI − 130.9 to 113.3; p = 0.89). Acute treatment with dexamethasone was associated with reductions of FGF21 by -175 pg/mL (95%CI − 236 to − 113; p < 0.001). Anti-inflammatory treatment with both, IL-1 antagonism and corticosteroids reduced FGF21 levels at short-term in individuals with the metabolic syndrome.

Trial registration: *ClinicalTrials.gov Identifiers* NCT02672592 and NCT00757276.

## Introduction

Fibroblast growth factor-21 (FGF21) is a central regulator of lipid, glucose, and energy homeostasis acting via the *β*Klotho-FGFR1 (fibroblast growth factor receptor 1) complex^1–3^. It is predominantly expressed in liver and adipose tissue and has shown to potently induce thermogenesis in brown adipose tissue in rodents^4–7^. Several studies have demonstrated that plasma levels of FGF21 are markedly elevated among patients with the metabolic syndrome^8–10^. Similarly, it is established that in patients with the metabolic syndrome inflammatory parameters are elevated due to the underlying chronic inflammation^11,12^. A correlation of FGF21 levels and inflammatory parameters has even been described^13,14,15^.

Indeed, in patients with metabolic syndrome, metabolic stress induces an activation of the innate immune system triggering a state of chronic low-grade inflammation^12,16,17^. In this context, IL-1β has been identified as a key mediator and has a causal role in the development of type 2 diabetes mellitus and of cardiovascular complications^18,19^. Recently, FGF21 has been proposed to link inflammation, insulin resistance and atherosclerosis^20,21^. We thus hypothesized that increased levels of FGF21 observed in patients with the metabolic syndrome are caused by an enhanced activity of inflammatory cytokines such as IL-1β. The aim of this study was to prove the principle that antiinflammatory treatment either via antagonism of the IL-1 pathway or via potent glucocorticoids would reduce FGF21 plasma levels.

Herein, we report the results of two clinical trials investigating the effects of short- and longer-term IL-1 antagonism as well as acute corticosteroid treatment on levels of FGF21 in obese patients with the metabolic syndrome.

## Methods

### Study design

This is a secondary analysis of two investigator-initiated, interventional trials involving patients with obesity and features of the metabolic syndrome, published previously^22,23^. Both trials were conducted according to the Declaration of Helsinki and the applicable International Conference on Harmonization guidelines on good clinical practice (ICH-GCP). The studies were approved by the local Ethics Committee northwest/central Switzerland (EKNZ) and legal authorities. Written informed consent was obtained from all patients. Both trials were preregistered on ClinicalTrials.gov (NCT02227420 and NCT02672592). Patients were recruited at two tertiary care centers in Switzerland (University Hospital Basel and Kantonsspital Aarau).

### Patients and study procedures of Trial A

Trial A was a prospective, open-labelled, interventional study involving 73 patients with obesity and at least one additional feature of the metabolic syndrome. The study procedure has been published in detail, previously^21^. In brief, inclusion criteria were age between 18 and 80 years, body-mass index (BMI) > 30 kg/m^2^ and at least one of the following additional features of the metabolic syndrome: hyperglycemia (HbA1c > 5.7%), hypertension (BP > 130/85 mmHg or blood pressure lowering therapy) or dyslipidemia (HDL < 1.0 mmol/l or triglycerides > 1.7 mmol/l or LDL > 3.4 mmol/l or lipid lowering treatment). Main exclusion criteria were concurrent medication with glucocorticoids, a diagnosis of Cushing’s syndrome, an underlying chronic inflammatory disease, a current infection or history of a severe infection within the previous 2 months, severe comorbidities such as hematologic, kidney or liver disease, cancer and pregnancy/breastfeeding.

Subjects were asked to have a standard dinner at latest 12 h before the study visits. All visits were scheduled early in the morning between 7.30 a.m. and 10 a.m. after an overnight fast. Apart from water, subjects fasted thereafter until the experiments were completed. Furthermore, study visits for every participant were consistently scheduled at the same time in order to exclude a confounding factor of circadian rhythm.

At baseline visit, clinical parameters were assessed and blood was drawn to measure baseline parameters. After baseline visit, an 1 mg overnight dexamethasone suppression test was performed according to the known standardized test procedures. Patients received a single pill of 1 mg dexamethasone to take orally at midnight and blood was collected at 8 a.m. the following morning after an overnight fast. After a washout period of 6 days all patients received three subcutaneous injections of the recombinant human interleukin-1-receptor antagonist anakinra/Kineret (100 mg, Swedish Orphan Biovitrum AB, Stockholm, Sweden) within 2 days. The consecutive injections were started at 8 p.m. and continued in 12 h’ time interval. After three injections blood was drawn to assess short-term effects of anakinra/Kineret. A complete description of the course of the study has been published earlier^23^.

### Patients and study procedures of Trial B

Trial B was a placebo-controlled, double-blinded, randomized intervention trial. Main eligibility criteria were similar to Trial A. Originally, the study had been designed to study effects of IL-1β antagonism on testosterone levels in men with the metabolic syndrome, therefore only male patients were included. All patients were aged 18 to 75 years with a BMI > 30 kg/m^2^ and total testosterone levels < 12 nmol/L. Complete eligibility criteria have been published recently^22^.

Patients were randomized 1:1 to either receive the study drug anakinra/Kineret or placebo as subcutaneous injections twice daily in a 12-h interval. Study visits were scheduled in the morning in a fasting state at baseline, at 1 day (visit 1) and at 4 weeks. Each subject was asked to abstain from alcohol and excessive physical exercise during the conduct of the study and to have a standard dinner at latest 12 h before the study visits.

### Laboratory analyses

Routine clinical laboratory parameters were measured at the central laboratories of both participating centers. C-reactive protein (CRP) was determined with an immunoturbidimetric assay (Tina-quant C-reactive Protein Gen. 3 Test, Roche Diagnostics GmbH, Mannheim, Germany). Blood samples were drawn and stored at − 80 °C to assess FGF21 in batch analysis. FGF21 was measured in serum samples using the human FGF21 Ella Simple Plex assay (ProteinSimple, San Jose, California, USA) which detects total FGF21 independent of plasmatic proteolytic N- or C-terminal cleavage^24^. The assay has a limit of detection of 3.74 pg/mL and an inter-assay coefficient of variation of 7.8% and an intra-assay coefficient of variation of 8.2%; mean values of three measurements were taken for analysis.

### Statistical analysis

Categorical variables are expressed as counts (percentage) and continuous variables as median (IQR) unless stated otherwise. We used ordinary least-squares linear regression model adjusted for the baseline value of the dependent variable to investigate changes in FGF21 levels. For analyses on associations of FGF21 levels with patient’s characteristics, comorbidities and clinical variables as well as short-term effects, data from both trials were pooled. Effects of glucocorticoids on FGF21 levels were assessed in Trial A. Short- and long-term effects of anakinra were assessed in Trial B. Pre-defined subgroup analyses with calculation of interaction terms were performed based on history of diabetes mellitus, and the occurrence of injection-site reactions. Statistical analyses were performed using STATA 14.2 (Stata Corp, College Station, TX, USA) and tests were done at a two-sided 5% significance level with two-sided 95% confidence intervals.

## Results

### Patient characteristics

Overall, 140 patients were included in this analysis, 73 from Trial A and 67 from Trial B. Baseline characteristics of patients are presented in Table 1. Median age was 55 years, and in Trial A 51% were female, whereas in Trial B all participants were male. Median BMI was 36.5 kg/m^2^ and pre-existing diabetes mellitus was more prevalent among patients in Trial A (64%) when compared to patients in Trial B (24%). Overall, the majority of trial participants suffered from hypertension (77% Trial A; 70% Trial B) and dyslipidemia (79% Trial A; 85% Trial B). Patients from Trial B had better glycemic control with an HbA1c of 5.8% as compared to 6.5% in patients from Trial A. Overall, included patients mirrored a state of chronic low-grade inflammation with a baseline CRP levels of 3.4 mg/L in both trials.

**Table 1:** Baseline characteristics and clinical variables of enrolled patients.

Study	Trial B (n = 67)		Trial A (n = 73)
Treatment	Placebo (n = 34)	Anakinra (n = 33)	Anakinra and dexamethasone
**General characteristics**			
Age—years	55 (43, 65)	55 (45, 67)	56 (48, 62)
Sex, female—%	0 (0%)	0 (0%)	37 (51%)
Body weight—kg	114 (102, 124)	113 (107, 122)	106 (96, 123)
Body mass index—kg/m^2^	36.2 (34.0, 39.5)	37.3 (34.4, 39.4)	36.3 (33.3, 40.7)
< 35	11 (32%)	10 (30%)	30 (41%)
35–40	18 (53%)	17 (52%)	19 (26%)
> 40	5 (15%)	6 (18%)	24 (33%)
Systolic blood pressure—mmHg	132 (123, 139)	136 (130, 147)	139 (129, 152)
Diastolic blood pressure—mmHg	85.3 (78, 94)	89.7 (84, 93)	85.0 (77, 91)
**Comorbidities**			
Diabetes mellitus	7 (21%)	9 (27%)	47 (64%)
Diet	0 (0%)	1 (11%)	5 (11%)
Oral drug	6 (86%)	8 (89%)	39 (83%)
Insulin (alone or with oral drug)	1 (14%)	0 (0%)	3 (6%)
Hypertension	21 (62%)	26 (79%)	56 (77%)
Antihypertensive medication	14 (67%)	16 (62%)	53 (91%)
Dyslipidemia	29 (85%)	28 (85%)	58 (79%)
Statin treatment	7 (24%)	11 (39%)	35 (58%)
Smoking status	19 (56%)	23 (70%)	38 (52%)
Packyears	7.5 (0.0, 20.0)	15.0 (0.0, 27.0)	0.0 (0.0, 35.0)
**Laboratory values**			
HbA1c—%	5.8 (5.4, 6.1)	5.9 (5.5, 6.3)	6.5 (5.8, 8.1)
Total cholesterol—mmol/L	4.7 (4.0, 5.5)	5.3 (3.8, 5.7)	4.6 (3.6, 5.3)
LDL cholesterol—mmol/L	2.8 (2.1, 3.8)	3.1 (1.8, 4.0)	2.4 (1.7, 3.1)
HDL cholesterol—mmol/L	1.1 (1.0, 1.3)	1.2 (1.0, 1.3)	1.1 (0.9, 1.4)
Triglycerides—mmol/L	1.8 (1.2, 2.6)	1.7 (1.3, 2.6)	1.8 (1.3, 2.4)
C-reactive protein—mg/L	3.6 (1.6, 4.9)	3.2 (2.1, 4.5)	3.4 (1.6, 6.6)
Interleukin-6—mg/L	2.2 (2.0, 3.9)	2.6 (2.0, 3.9)	3.7 (1.6, 6.8)

Data are presented as median (IQR) or n (%). HDL, high-density lipoprotein; LDL, low-density lipoprotein. Concentrations of Interleukin-6 in the two studies were measured with different assays.

### Associations of FGF21 levels with demographic characteristics, comorbidities and clinical variables

Baseline levels of FGF21 were significantly higher among diabetics when compared to non-diabetics and FGF21 levels increased in a linear fashion by 84 pg/mL (95%CI 47 to 121; p < 0.001) with each percentage point increase in HbA1c. Accordingly, glycemic parameters such as HOMA-index, insulin, C-peptide and fasting glucose levels were strongly correlated with FGF21 levels at baseline (Table 2). Patients on statins also featured significantly higher FGF21 levels compared to patients without such medication. Each stepwise mg per L increase in CRP levels was associated with higher baseline FGF21 values by 15 pg/mL (95% CI 5 to 26; p = 0.004). Likewise, each stepwise pg per mL increase in Interleukin-6 (IL-6) concentration was associated with higher baseline FGF21 values by 16 pg/mL (95% CI 4 to 28; p = 0.01) (Table 2).

**Table 2:** Association of baseline FGF21 levels with demographic characteristics, comorbidities and clinical variables.

	Regression analysis coefficient (95% CI)	*p* value
**General characteristics**		
Age at study entry—years	1.75 (− 3.05, 6.57)	0.47
Female sex	− 8.85 (− 140.91, 123.21)	0.89
Smoking status	− 80.06 (− 196.96, 36.85)	0.18
Packyears—years	0.84 (− 1.70, 3.38)	0.65
**Clinical parameters**		
Body weight—kg	− 1.57 (− 4.96, 1.83)	0.36
BMI—kg/m^2^	2.92 (− 9.17, 15.02)	0.48
Waist circumference—cm	0.03 (− 5.93, 5.99)	0.99
Body surface area—m^2^	− 166.31 (− 434.58, 101.97)	0.22
Systolic blood pressure—mmHg	4.16 (0.56, 7.76)	0.02
Diastolic blood pressure—mmHg	3.07 (− 2.75, 8.89)	0.29
**Comorbidities**		
Prediabetes	120.72 (− 66.76, 308.19)	0.20
Diabetes mellitus	150.63 (38.42, 266.83)	0.009
Insulin treatment	236.03 (99.13, 372.93)	0.001
Hypertension	131.31 (1.10, 261.51)	0.048
Antihypertensive treatment	151.73 (− 21.22, 324.69)	0.09
Dyslipidemia	94.51 (56.71, 245.72)	0.22
Statin treatment	168.50 (38.19, 298.00)	0.01
Obstructive sleep apnea	44.83 (− 73.79, 163.47)	0.46
**Laboratory variables**		
HbA1c—%	83.62 (46.56, 120.66)	< 0.001
Fasting glucose—mmol/L	86.44 (35.39, 137.48)	0.001
Insulin—mmol/L	2.05 (0.35, 3.75)	0.02
C peptide—mmol/L	0.13 (0.04, 0.21)	0.006
logHOMA index	131.33 (32.21, 230.45)	0.01
C-reactive protein—mg/L	15.42 (4.97, 25.86)	0.004
Interleukin-6—pg/mL	15.98 (3.87, 28.09)	0.01

All laboratory values were obtained after overnight fast. Due to skewed distribution, HOMA-index was log-transformed.

### Short and longer-term effects of anakinra on FGF21

**Figure 1:**
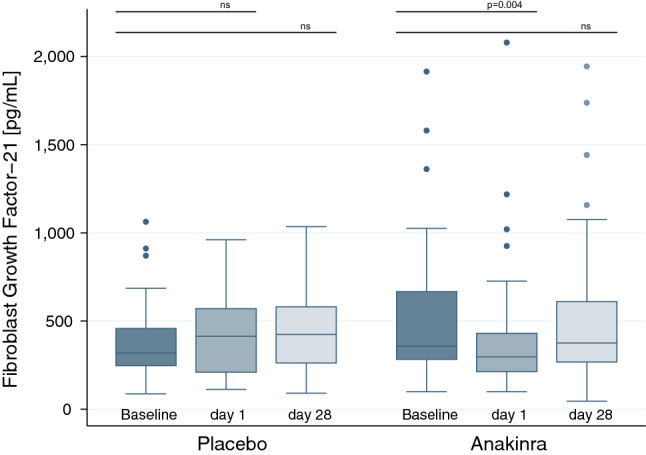
Box plots of FGF21 levels at baseline (dark blue) and after short-term treatment (medium blue) as well as after longer-term treatment (light blue) in patients randomized to placebo or anakinra from Trial B, respectively. P-values were determined using a linear mixed model. Each box signifies the upper and lower quartiles, while the median is represented by a line within the box. Whiskers represent the upper and lower adjacent values, outliers are depicted as dots.

At baseline, FGF21 levels were similar in both groups (placebo: 319 pg/mL [IQR 247–458], anakinra 357 pg/mL [IQR 283–666]; p = 0.15). Randomized treatment with anakinra significantly reduced in FGF21 levels after 1 day as compared to placebo, resulting in a between-group difference of − 200 pg/mL (95%CI − 334 to − 66 pg/mL; p = 0.004). At 4 weeks, this effect was no longer visible. Over the treatment period of 28 days, FGF21 levels increased in both groups, yielding a non-significant between-group difference of − 12 pg/mL (95%CI − 177 to 153 pg/mL; p = 0.89) (Fig. 1). When stratified by history of diabetes, there was no subgroup difference between diabetics and non-diabetics (p = 0.71). Longer-term treatment with anakinra was associated with injection-site reactions in 20 patients (60%) which appeared between days 7 and 22 and dis-appeared between days 15 and 30. The severity of these injection-site reactions was described as either mild or moderate, respectively.

### Effects of glucocorticoids on FGF21

**Figure 2:**
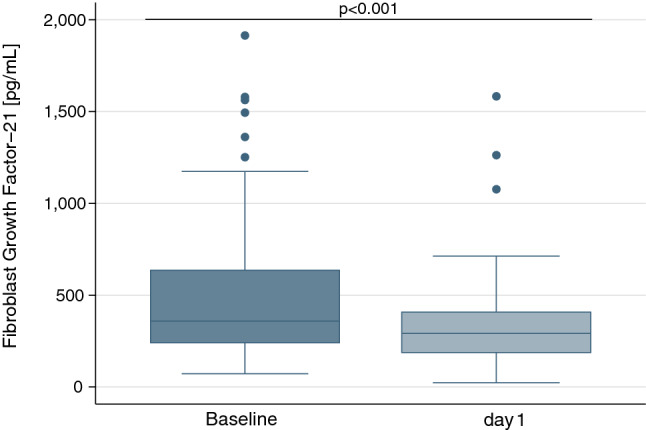
Box plots of FGF21 levels at baseline (blue) and after short-term treatment with dexamethasone (light blue) for patients from Trial A (n = 73). P-values were determined by Wilcoxon signed-rank test. Each box signifies the upper and lower quartiles, while the median is represented by a line within the box. Whiskers represent the upper and lower adjacent values; outliers are depicted as dots.

**Figure 3:**
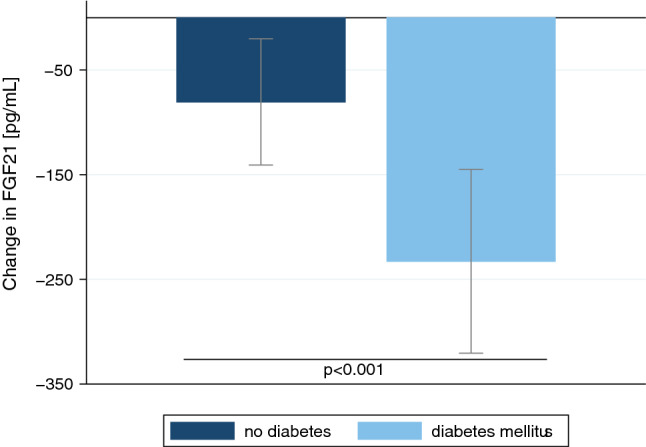
Effects of dexamethasone on serum FGF21 levels. Bar graph with mean absolute differences in FGF21 levels (± 95% confidence interval) between the baseline value and measurements at day 1 in patients without (blue) and with diabetes mellitus (light blue). P-value determined by linear regression stratified by the history of diabetes mellitus included as interaction term.

Overnight treatment with 1 mg dexamethasone significantly reduced median FGF21 levels from 359 pg/mL (IQR 240 to 636 pg/mL) to 292 pg/mL (IQR 187 to 408 pg/mL), yielding an average absolute difference from baseline of − 175 pg/mL (95%CI − 236 to − 113 pg/mL; p < 0.001) (Fig. 2). Patients with diabetes had a more pronounced decrease in FGF21 levels after dexamethasone, as compared to patients without diabetes (− 233 pg/mL [95%CI − 322 to − 144 pg/mL] vs. − 80 pg/mL [95%CI − 143 to − 18 pg/mL]; p < 0.001) (Fig. 3).

## Discussion

The key findings of this study are three-fold: First, among obese patients with features of the metabolic syndrome, levels of FGF21 were markedly elevated and correlated with markers of chronic low-grade inflammation and insulin resistance. Second, short-term anti-inflammatory treatment with an interleukin-1 antagonist transiently reduced FGF21 levels. Third, short-term treatment with glucocorticoids likewise diminished systemic levels of FGF21.

Previous studies have demonstrated that plasma FGF21 concentrations are increased in metabolic syndrome and correlate with insulin resistance^9^. Conversely, serum concentrations of FGF21 have been shown to be lower in patients with anorexia nervosa and to increase with weight gain^25^. In our two study cohorts we observed no correlation between FGF21 levels and body weight or waist circumference, but rather to markers of metabolic dysregulation and most profoundly to markers of inflammation.

In fact, there is increasing evidence suggesting that systemic inflammation plays a major role on the release of circulating FGF21. The secretion of FGF21 has been found to be stimulated upon acute inflammatory stimuli, such as lipopolysaccharide^26^. Likewise, states of (per)acute systemic inflammation such as systemic inflammatory response syndrome and sepsis have been shown to be associated with profound elevations of serum FGF21^27–29^. In our study, we observed a strong correlation of FGF21 with CRP. This observation led to the assumption that FGF21 acts as an acute phase protein, modulating inflammatory response. In fact there is increasing evidence pointing towards direct interactions of FGF21 with immune cell responses. For instance, FGF21 has been shown to mediate glucose utilization in monocytes which is highly important for their activation in inflammatory responses^30^. In experimental models of atherosclerosis, FGF21 has been demonstrated to exert anti-atherosclerotic effects by a reduction of NLRP3 related pryoptosis of endothelial cells^31^. Furthermore, there is evidence that FGF21 regulates T-cell development in the thymus^32^.

Experimental data support the notion that the inflammatory cytokine IL-1β impacts on the expression and signaling of β-Klotho^33^—a relevant coreceptor of FG21 that regulates downstream signaling pathways and exerts metabolic effects^34,35^. As a consequence, we postulated that antagonism of the IL-1β inflammatory pathway would lower FGF21 levels in patients with the metabolic syndrome. Indeed, in two independent studies we could show that treatment with the IL-1 receptor antagonist anakinra led to reduced FGF21 levels on the short-term, however, without significant effect after prolonged treatment. While the data from these trials do not give a clear indication on the underlying cause, the lack of long-term effect might primarily be due to impaired efficacy of IL-1 antagonism. Injection-site reactions were frequently observed after 1 week of treatment, but were absent in the short-term study. It is well established that the development of injection-site reactions is associated with inflammation in the subcutaneous tissue, with consecutive local sequestration of IL-1Ra, diminishing the effects of IL-1 antagonism as we^36^ and others^37,38^ observed in previous studies. It is therefore possible that longer-term effects of anakinra treatment on FGF21 levels may have been attenuated by the high percentages of patients developing injection-site reactions. Alternatively, compensatory elevations of other cytokines such as tumor necrosis factor-α (TNF-α) may as well explain temporary effects of anakinra. TNF-α is known to be another key mediator of inflammation in adipose tissue and experimental data demonstrate that it modulates FGF21 action^39^. Obviously, further studies investigating longer-term anti-inflammatory treatment are needed—ideally with a drug causing less injection site reactions than anakinra, e.g. canakinumab.

FGF21 has proven anti-inflammatory action mediated via inhibition of the activation of nuclear factor-κB, almost similar to the effects of glucocorticoids^40–42^. Elevations of FGF21 in inflammatory states might therefore be counterregulatory. In fact, in a previous study on patients with acute inflammatory response syndrome due to community-acquired pneumonia, FGF21 strongly correlated with the severity of pneumonia. In this study, treatment with prednisone over 7 days led to a significant reduction of acutely increased FGF21 levels when compared with placebo^28^. To date, effects of glucocorticoids on chronically elevated FGF21 in metabolically ill patients are unknown.

In this study, we found a similar effect with pronounced suppression of systemic FGF21 levels in response to a single dose treatment with dexamethasone. In contrast, a study on patients with chronic endogenous hypercortisolemia due to Cushing’s syndrome did not find any effects of cortisol on serum FGF21. Among those patients, levels of FGF21 were significantly elevated compared to lean controls, however not different from obese patients without Cushing’s syndrome^43^. Above data would suggest that there might be a differential regulation of FGF21 in response to acute versus chronic exposure to glucocorticoids or IL-1 antagonism as well as dose and potency of glucocorticoids. However, this hypothesis needs to be further explored in future studies.

In contrast, in rodents glucocorticoids have shown to induce FGF21 expression in the liver in a feed-forward mechanism by peroxisome proliferator-activated receptor-α^44^. Furthermore, in experimental studies chronic treatment with glucocorticoids likewise triggered both production and secretion of FGF21, at least in the experimental setting^45,46^. However, it is now established that the metabolic role of FGF21 seems to be fundamentally different between humans and rodents and therefore data from rodent studies cannot be extrapolated on humans^35^.

Both, IL-1 antagonism and glucocorticoids led to reduced circulating levels of FGF21 in our trials. While it is known that FGF21 can be cleaved proteolytically at both the N terminus and the C terminus^47,48^, an enhanced proteolytic degradation seems unlikely since the used assay detects the total amount of FGF21 independent of cleavage. For this reason, we hypothesize that a reduced hepatic production or secretion may be the main effect of both treatments. However, an additional effect on the cleavage and inactivation of FGF21 cannot be excluded with our data.

Data on circadian oscillations of FGF21 are conflicting with some studies demonstrating the absence of any diurnal variation in healthy subjects^49^, while other studies reported its presence^50^. Due to the standardized protocols in our studies without variations in diet, exercise or time of visits, such confounding factors were minimized.

Following strengths of our study are noteworthy: Validation of the findings in two independent interventional trials with well-defined patient cohorts, and the use of robust and precise measurement methods. Furthermore, this is, to the best of our knowledge, the first placebo-controlled randomized trial investigating the effect of IL-1 antagonism on FGF21 levels. Naturally, our study has some limitations. First, this is a secondary analysis of two studies, which were primarily not designed and powered to show effects on FGF21, in particular for the longer-term results. Second, patients included in the trials presented only marginal systemic inflammation and rather high frequency of injection-site reactions, which may have led to an underestimation of the effects of anti-inflammatory treatment on FGF21.

In conclusion, our results show a transient decrease in FGF21 levels upon treatment with IL-1 antagonism and with a single dose of dexamethasone. Whether there is a longer-lasting effect has to be investigated in future studies. Further studies are needed to understand the potential role of FGF21 in systemic inflammation and metabolic stress.

## References

[1] Angelin B, Larsson TE, Rudling M (2012). Circulating fibroblast growth factors as metabolic regulators—A critical appraisal. Cell Metab..

[2] Inagaki T (2007). Endocrine regulation of the fasting response by PPARα-mediated induction of fibroblast growth factor 21. Cell Metab..

[3] Potthoff MJ (2009). FGF21 induces PGC-1α and regulates carbohydrate and fatty acid metabolism during the adaptive starvation response. Proc. Natl. Acad. Sci. USA..

[4] Chau MDL, Gao J, Yang Q, Wu Z, Gromada J (2010). Fibroblast growth factor 21 regulates energy metabolism by activating the AMPK-SIRT1-PGC-1α pathway. Proc. Natl. Acad. Sci. USA..

[5] Hanssen MJW (2015). Serum FGF21 levels are associated with brown adipose tissue activity in humans. Sci. Rep..

[6] Lee P (2013). Mild cold exposure modulates fibroblast growth factor 21 (FGF21) diurnal rhythm in humans: Relationship between FGF21 levels, lipolysis, and cold-induced thermogenesis. J. Clin. Endocrinol. Metab..

[7] Lee P (2014). Irisin and FGF21 are cold-induced endocrine activators of brown fat function in humans. Cell Metab..

[8] Barb D, Bril F, Kalavalapalli S, Cusi K (2019). Plasma fibroblast growth factor 21 is associated with severity of nonalcoholic steatohepatitis in patients with obesity and type 2 diabetes. J. Clin. Endocrinol. Metab..

[9] Chavez AO (2009). Circulating fibroblast growth factor-21 is elevated in impaired glucose tolerance and type 2 diabetes and correlates with muscle and hepatic insulin resistance. Diabetes Care.

[10] Ebert T (2018). Relationship between 12 adipocytokines and distinct components of the metabolic syndrome. J. Clin. Endocrinol. Metab..

[11] Ballak DB, Stienstra R, Tack CJ, Dinarello CA, van Diepen JA (2015). IL-1 family members in the pathogenesis and treatment of metabolic disease: Focus on adipose tissue inflammation and insulin resistance. Cytokine.

[12] Donath MY, Dalmas É, Sauter NS, Böni-Schnetzler M (2013). Inflammation in obesity and diabetes: Islet dysfunction and therapeutic opportunity. Cell Metab..

[13] Kralisch S (2013). Fibroblast growth factor-21 serum concentrations are associated with metabolic and hepatic markers in humans. J. Endocrinol..

[14] Novotný D (2014). Evaluation of total adiponectin, adipocyte fatty acid binding protein and fibroblast growth factor 21 levels in individuals with metabolic syndrome. Physiol. Res..

[15] Reinehr T (2016). Inflammatory markers in obese adolescents with type 2 diabetes and their relationship to hepatokines and adipokines. J. Pediatr..

[16] Cottam DR (2004). The chronic inflammatory hypothesis for the morbidity associated with morbid obesity: Implications and effect of weight loss. Obes. Surg..

[17] Ridker PM, Wilson PWF, Grundy SM (2004). Should C-reactive protein be added to metabolic syndrome and to assessment of global cardiovascular risk?. Circulation.

[18] Dinarello CA (2011). Interleukin-1 in the pathogenesis and treatment of inflammatory diseases. Blood.

[19] Donath MY, Shoelson SE (2011). Type 2 diabetes as an inflammatory disease. Nat. Rev. Immunol..

[20] Kim KH (2013). Autophagy deficiency leads to protection from obesity and insulin resistance by inducing Fgf21 as a mitokine. Nat. Med..

[21] Tabari FS (2019). The roles of FGF21 in atherosclerosis pathogenesis. Rev. Endocr. Metab. Disord..

[22] Ebrahimi F (2018). IL-1 antagonism in men with metabolic syndrome and low testosterone: A randomized clinical trial. J. Clin. Endocrinol. Metab..

[23] Urwyler SA, Schuetz P, Ebrahimi F, Donath MY, Christ-Crain M (2017). Interleukin-1 antagonism decreases cortisol levels in obese individuals. J. Clin. Endocrinol. Metab..

[24] Zhen EY, Jin Z, Ackermann BL, Thomas MK, Gutierrez JA (2016). Circulating FGF21 proteolytic processing mediated by fibroblast activation protein. Biochem. J..

[25] Dostálová I (2008). Plasma concentrations of fibroblast growth factors 19 and 21 in patients with anorexia nervosa. J. Clin. Endocrinol. Metab..

[26] Feingold KR (2012). FGF21 is increased by inflammatory stimuli and protects leptin-deficient ob/ob mice from the toxicity of sepsis. Endocrinology.

[27] Gariani K, Drifte G, Dunn-Siegrist I, Pugin J, Jornayvaz FR (2013). Increased FGF21 plasma levels in humans with sepsis and SIRS. Endocr. Connect..

[28] Ebrahimi F (2019). Fibroblast growth factor 21 predicts outcome in community-acquired pneumonia: Secondary analysis of two randomised controlled trials. Eur. Respir. J..

[29] Thiessen SE, Vanhorebeek I, Derese I, Gunst J, Van Den Berghe G (2015). FGF21 response to critical illness: Effect of blood glucose control and relation with cellular stress and survival. J. Clin. Endocrinol. Metab..

[30] Wang, N. *et al.* FGF-21 Plays a Crucial Role in the Glucose Uptake of Activated Monocytes. 10.1007/s10753-017-0665-7. 10.1007/s10753-017-0665-7 28965199

[31] Zeng Z (2020). FGF21 mitigates atherosclerosis via inhibition of NLRP3 inflammasome-mediated vascular endothelial cells pyroptosis. Exp. Cell Res..

[32] Nakayama Y, Masuda Y, Ohta H, Tanaka T, Washida M, Nabeshima YI, Miyake A, Itoh N, Konishi M (2017). Fgf21 regulates T-cell development in the neonatal and juvenile thymus. Sci. Rep..

[33] Zhao Y (2016). IL-1β inhibits β-Klotho expression and FGF19 signaling in hepatocytes. Am. J. Physiol. Endocrinol. Metab..

[34] Fisher FM, Maratos-Flier E (2016). Understanding the physiology of FGF21. Annu. Rev. Physiol..

[35] Staiger H, Keuper M, Berti L, de Angelis MH, Häring HU (2017). Fibroblast growth factor 21-metabolic role in mice and men. Endocr. Rev..

[36] Ebrahimi F (2019). Effects of interleukin-1 antagonism on cortisol levels in individuals with obesity: A randomized clinical trial. Endocr. Connect..

[37] Kaiser C (2012). Injection-site reactions upon Kineret (anakinra) administration: Experiences and explanations. Rheumatol. Int..

[38] Van Asseldonk EJP (2011). Treatment with Anakinra improves disposition index but not insulin sensitivity in nondiabetic subjects with the metabolic syndrome: A randomized, double-blind, placebo-controlled study. J. Clin. Endocrinol. Metab..

[39] Díaz-Delfín J (2012). TNF-α represses β-klotho expression and impairs FGF21 action in adipose cells: Involvement of JNK1 in the FGF21 pathway. Endocrinology.

[40] Yu Y (2016). Fibroblast growth factor 21 (FGF21) inhibits macrophage-mediated inflammation by activating Nrf2 and suppressing the NF-κB signaling pathway. Int. Immunopharmacol..

[41] Yu Y (2015). Fibroblast growth factor 21 (FGF21) ameliorates collagen-induced arthritis through modulating oxidative stress and suppressing nuclear factor-kappa B pathway. Int. Immunopharmacol..

[42] Zhang Y, Liu Z, Zhou M, Liu C (2018). Therapeutic effects of fibroblast growth factor-21 against atherosclerosis via the NF-κB pathway. Mol. Med. Rep..

[43] Ďurovcová V (2010). Plasma concentrations of fibroblast growth factors 21 and 19 in patients with Cushing’s syndrome. Physiol. Res..

[44] Patel R (2015). Glucocorticoids regulate the metabolic hormone FGF21 in a feed-forward loop. Mol. Endocrinol..

[45] Al-Aqil FA (2018). Interaction of glucocorticoids with FXR/FGF19/FGF21-mediated ileum-liver crosstalk. Biochim. Biophys. Acta Mol. Basis Dis..

[46] Vispute SG, Bu P, Le Y, Cheng X (2017). Activation of GR but not PXR by dexamethasone attenuated acetaminophen hepatotoxicities via Fgf21 induction. Toxicology.

[47] Micanovic R (2009). Different roles of N- and C-termini in the functional activity of FGF21. J. Cell. Physiol..

[48] Dunshee DR (2016). Fibroblast activation protein cleaves and inactivates fibroblast growth factor 21. J. Biol. Chem..

[49] Gälman C (2008). The circulating metabolic regulator FGF21 is induced by prolonged fasting and PPARα activation in man. Cell Metab..

[50] Yu H (2011). Circadian rhythm of circulating fibroblast growth factor 21 is related to diurnal changes in fatty acids in humans. Clin. Chem..

